# Integration of art and technology in personalized radiation oncology care: Experiences, evidence, and perspectives

**DOI:** 10.3389/fpubh.2023.1056307

**Published:** 2023-01-23

**Authors:** Calogero Casà, Loredana Dinapoli, Elisa Marconi, Silvia Chiesa, Patrizia Cornacchione, Francesco Beghella Bartoli, Serena Bracci, Alessandra Salvati, Sara Scalise, Giuseppe Ferdinando Colloca, Daniela Pia Rosaria Chieffo, Maria Antonietta Gambacorta, Vincenzo Valentini, Luca Tagliaferri

**Affiliations:** ^1^UOC di Radioterapia Oncologica, Fatebenefratelli Isola Tiberina, Gemelli Isola, Rome, Italy; ^2^UOC di Radioterapia Oncologica, Dipartimento Diagnostica per Immagini, Radioterapia Oncologica ed Ematologia, Fondazione Policlinico Universitario Agostino Gemelli IRCCS, Rome, Italy; ^3^UOS di Psicologia Clinica, Fondazione Policlinico Universitario Agostino Gemelli IRCCS, Rome, Italy; ^4^Dipartimento di Scienze Radiologiche ed Ematologiche Università Cattolica del Sacro Cuore, Rome, Italy; ^5^Scienze della Salute della Donna, del Bambino e di Sanità Pubblica Università Cattolica del Sacro Cuore, Rome, Italy

**Keywords:** radiotherapy, technology, art, digital, personalization, oncology, engagement, patient-centered care

## Abstract

Cancer diagnoses expose patients to traumatic stress, sudden changes in daily life, changes in the body and autonomy, with even long-term consequences, and in some cases, to come to terms with the end-of-life. Furthermore, rising survival rates underline that the need for interventions for emotional wellbeing is in growing demand by patients and survivors. Cancer patients frequently have compliance problems, difficulties during treatment, stress, or challenges in implementing healthy behaviors. This scenario was highlighted during the COVID-19 emergency. These issues often do not reach the clinical attention of dedicated professionals and could also become a source of stress or burnout for professionals. So, these consequences are evident on individual, interpersonal, and health system levels. Oncology services have increasingly sought to provide value-based health care, considering resources invested, with implications for service delivery and related financing mechanisms. Value-based health care can improve patient outcomes, often revealed by patient outcome measures while seeking balance with economical budgets. The paper aims to show the Gemelli Advanced Radiation Therapy (ART) experience of personalizing the patients' care pathway through interventions based on technologies and art, the personalized approach to cancer patients and their role as “co-stars” in treatment care. The paper describes the vision, experiences, and evidence that have guided clinical choices involving patients and professionals in a co-constructed therapeutic pathway. We will explore this approach by describing: the various initiatives already implemented and prospects, with particular attention to the economic sustainability of the paths proposed to patients; the several pathways of personalized care, both from the patient's and healthcare professional perspective, that put the person's experience at the Gemelli ART Center. The patient's satisfaction with the treatment and economic outcomes have been considered. The experiences and future perspectives described in the manuscript will focus on the value of people's experiences and patient satisfaction indicators, patients, staff, and the healthcare organization.

## 1. Introduction: Integration of art and technology in personalized radiation oncology care

Cancer diagnosis exposes patients to traumatic stress, and sudden changes in daily life, in body and autonomy, with even long-term consequences; some of them have to deal with the end-of-life phase (1). Over the last three decades, cancer mortality has shown an important decline, especially in most high-income countries, reflecting improvements in cancer prevention, diagnosis, and management (2). The rising of survival rates underlines also a growing need for interventions to improve patients' emotional wellbeing (2). As a fact, cancer patients frequently have difficulties during treatment, such as anxiety, depression, and stress (3, 4). These issues affect adherence to treatment, cancer survival, and treatment costs (5). Among oncological treatments, radiotherapy (RT) requires an everyday burden, often worsened by concomitant chemotherapy (4, 6, 7). The duration of treatments and the possible side effects are challenging for every oncological patient (8, 9). These are long-term treatments performed mainly on an outpatient basis, often making patients prone to show emotional discomfort (4, 6, 7).

During the last years the importance of complementary psychological support therapies in alleviating cancer patients' distress, depression as well as fatigue, and pain, has been demonstrated (10–12).

Among creative therapies, art therapy is assuming an increasingly important role in improving communication, awareness, and patient quality of life (QoL) (13). Art therapy is a form of psychotherapy that uses the expressive qualities of the visual sign in the context of a therapeutic relationship. This is meant to bring about personal change to increase wellbeing and psychological functioning (14–16). A recent systematic review (17) highlighted that, even if the mechanisms of these beneficials are still unclear, art therapy is motivating, deepens understanding, insight, and mastery, and also provides a safe and structured pathway for self-awareness (17). It also alleviates physical suffering and improves coping skills by increasing feelings of energy (15, 18). Of course, art therapy does not replace standard medical treatments. Rather, it should be integrated into a personalized, multidisciplinary approach that recognizes the role of the mind in influencing the body, promoting wellbeing and stimulating coping skills in stressful situations.

Nowadays, a significant change is taking place highlighting a patient-centered care pathway instead of a discipline-centered one (19, 20). In this scenario, several initiatives have been developed to improve patient-centeredness in cancer care (21, 22). In particular, strategies have been adopted worldwide to ensure respect for patient preferences, emotional support, physical comfort, information/communication needs, care coordination, family and friends' involvement, and access to care (23).

According to Gemelli ART (Advanced Radiation Therapy) experience, we believe that cancer patients' deepest and often unrevealed aspirations are to be cared for. Each patient is welcomed as a person living the experience of disease, potentially disabling body and soul. Technology is a tool that, under the guidance of the knowledge and expertise of our Center professionals, focuses on meeting those needs: treating the patient and taking care of the person. Thanks to the use of artificial intelligence (AI) and advanced medical technologies, it is increasingly possible to realize personalized and tailored cancer care pathways (24, 25). In our context, we are investigating the combination of art and technology to include patients' emotional and relational experiences. At this stage, we are studying how to implement at our best interventions based on art and technology on patients, staff and caregivers and how this implementation could impact their sense of involvement and care satisfaction. These dimensions often have an impact on treatment and care compliance. The impact of these variables on treatment tolerance and other clinical outcomes could be investigated in the future.

This article then aims to report the experience of our Center, where a large number of interventions that merge art and technology have been undergoing for 10 years already. This experience systematically integrates digital technology and the beauty of art into the basic standard of cancer care to provide an holistic answer to cancer patients clinical and human needs. If “Value-based medicine” is defined as the “practice that incorporates the highest level of evidence-based data with the patient-perceived value conferred by health care interventions for the resources expended” (4–6), we strive for such integration of art, technology and patient needs to lead to better personalization of treatments and, where possible, also to a positive economic impact on the National Health System.

## 2. Art, creativity, and technologies in cancer treatment: The Italian experience of Gemelli ART

In the following section, according to the template of the approach described above, we will describe some interventions that took place in our Center over time, involving patients and staff. Some of these projects have become multi-center experiences. The Gemelli ART Radiation Oncology department provides patients with technologically advanced instruments (ART as Advanced Radiation Therapy) and a multidisciplinary team. This center is made up of an Operating Sector with RT bunkers (4 cone-beam CT linear accelerators, 1 MRI-linear accelerators), an Interventional Oncology Center for Interventional Radiotherapy (brachytherapy), outpatient clinics for medical visits and psychological support service, a Day Hospital and two Inpatient wards. Gemelli ART, however, also stands for Art because it involves welcoming environments, exclusively decorated therapy rooms and a stunning mosaic that enriches patient's journey within our Center and offers relief through the beauty of art.

***2017 - The value of patient experience***

### 2.1. Patient's satisfaction in quantitative measures in the RAMSI project

Following the slogan: “*Technology at the service of knowledge, knowledge at the service of the patient,”* the aim is to provide healthcare services based on dynamic mechanisms focused on the patient and the quality of services. Improving healthcare quality is often reflected in clinical outcomes and patient satisfaction but it also has to consider the costs of the services offered. Patient satisfaction is recognized as a key performance indicator for monitoring the quality of hospitals (26). Through systematic analysis of patient-relevant data, decision-making processes can be tailored to patients, empowering them to engage with healthcare systems, maximizing their health and wellbeing, and thus minimizing attrition (27).

**Figure 1:**
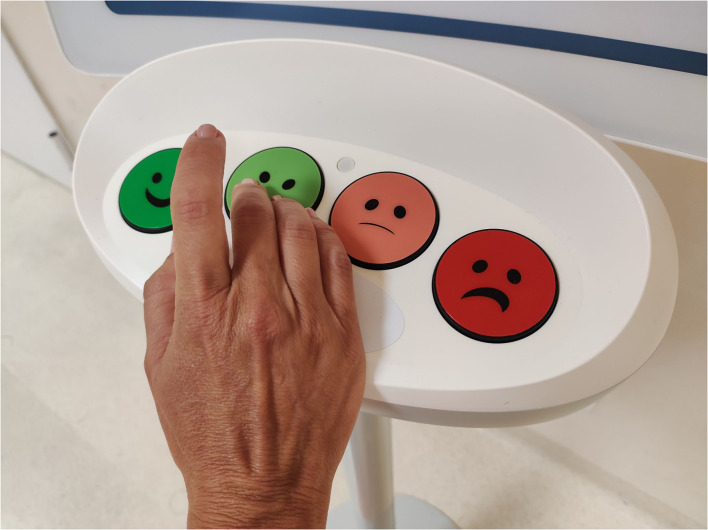
RAMSI project totem.

The RAMSI *Radioterapia Amica Mia* (Radiotherapy My Friend) *Smile In*™ (SI) project has foreseen the placement of SI totems with four push buttons using the HappyOrNot technology (RetailIN, Cesano Maderno MB, Italy: https://smilein.it) in our RT department. It has enabled the collection and analysis of patient feedback in the form of self-reported experience in real-time (27). Physical SI totems were installed in places of greatest affluence to promptly detect patients input and collect data on their experience during RT using HappyOrNot technology. Specifically, these locations were identified as: waiting rooms for clinics and treatment rooms, the access points and exit from the treatment rooms, and the RT service. Patients read the allocated question in the question sheet holder and gave their feedback anonymously by touching a smiley button (Figure 1). Four different faces define four assessment points: “very positive,” “positive,” “negative,” and “very negative”. To assess patient's needs and experiences, four areas of interest were defined:

- Patient-centric welcome perception: The perception of human and environmental welcome during clinics and treatments;
- Punctuality: Visits and treatments time adherence to planned schedules;
- Professionalism: Healthcare workers' competence or skill expected;
- Comfort: Environmental and human capability to accomplish patients' needs.

The RAMSI project effectively puts the patients at the center of the therapeutic process as a person in their complexity to preserve their QoL and human dignity during the radiation treatment. Furthermore, it provides a fast, easy-to-use tool to extract patient satisfaction data.

### 2.2. Patients' quality reports: The HAPPY protocol (Humanity Assurance Protocol in interventional radiotheraPY)

In clinical settings, as well as in oncological field, the decision-making process of treatment pathway is based on the interaction between physician and patient. However, cultural factors influence patients perceptions of the disease and treatment choices, often conditioned by factors such as age, socioeconomic status, education level, language, geographical area of origin (urban or rural), spirituality, sexual orientation, or occupation (28, 29).

The patient's psychological state is often added to the context described above. Often, anxiety and depression can reduce compliance with treatment and affect the clinical outcome (30, 31).

Based on that evidence, our Interventional RT Department proposed a study to investigate the lack of knowledge regarding needs and expectations of gynecological cancer patients and to hypothesize solutions to improve patients' emotional state and sensitivity.

To achieve this objective, the importance of each professional figure who comes into contact with the patient during the therapeutic pathway was considered, as each staff member can contribute to improving patient's management. Among the figures involved we can find physician, considering the importance of providing clinical Information for the management of symptoms, the psychologist to provide psychological support, nurses and Radiation Therapy Technologist (RTT) for the reception and reassurance of patients during treatment. The whole team contributes significantly in helping patient to better cope with the disease (20, 32–37).

We examined needs, values, expectations, and preferences among gynecological cancer patients. A specific focus was dedicated to communication and the need for information regarding therapy efficacy, side effects, and toxicities, analyzing collected data to generate working hypotheses. The second objective of this work was to propose a series of interventions/recommendations to ensure a sensitive approach to fostering the patient's psychological wellbeing during interventional Radiotherapy.

The project, which considered a sample of 30 gynecological cancer patients, was conceived and carried out within the study group of brachytherapy, interventional Radiotherapy, and intra-operative Radiotherapy (IORT) of the Italian Association of Radiotherapy and Clinical Oncology (AIRO Associazione Italiana di Radioterapia ed Oncologia Clinica).

A multi-professional team was chosen to assess the needs using a multidimensional approach composed of 1 interventional radiologist, one geriatric oncologist, one nurse, one psychologist, one radio-oncology resident, and 1 RTT. Each member of the multi-professional team performed several independent multidimensional conversations with the patients. Each patient had six different discussions, for a total of 180 talks. After this phase, the multi-professional team scheduled two meetings, the first to collect all the needs coming from the patients and the second to finalize the classification by selecting the most represented needs as a result of the 180 multidimensional conversations.

The results of the task group were submitted to an Expert Team of four physicians from 4 different institutions for a final evaluation. Both teams discussed patients needs to generate a list of interventions/recommendations aimed to address each individual need to achieve their inner wellbeing. Finally, a Master Team carried out an independent check of the project and approved it.

The list of interventions identified was HAPPY (Humanity Assurance Protocol in interventional radiotheraPY) and consists of a protocol that can be exported to other centers to guarantee humanity and the best quality of care and compliance to treatments.

Among the recommendations highlighted there is the possibility of using simple language or alternatives to terms such as brachytherapy or bunkers, which can cause more significant anxiety, as well as the possibility of creating a more welcoming hospital environment with colors or images designed to ensure a warmer and more familiar territory. Music therapy can also help manage anxiety, as favorite music can stimulate the relaxation response by activating the parasympathetic system, restoring the balance of the autonomic nervous system (38, 39).

***2018 - Customized “targets” for young patients***

### 2.3. Psychological, art, and digital interventions for pediatrics: The RADAR project

Special attention has been paid overtime to young patients. In pediatric RT, obtaining the cooperation necessary for the preparation and administration of treatment is particularly complicated, as it is challenging for a child to stand still and alone (40, 41). When patients are unable to maintain a fixed and reproducible position (42), sedation or general anesthesia (GA) becomes necessary (43). In general, RT children and adolescents undergo several changes in their lifestyle (41), daily, school, and social activities (44); changes and stress are more significant in the case of GA, also due to fasting. Furthermore, the use of GA may increase the risk of medical complications (45) and impact healthcare costs (45, 46). Different studies have described the benefit of combining psychological support interventions with standard therapies to reduce the number of sedations (45, 47, 48). It has been demonstrated that a multidisciplinary approach implemented by a specialized team (49) can identify patient's needs and allow targeted interventions to facilitate treatment preparation and improve patient compliance, thus avoiding sedation when possible (46, 50–54). There are many different interventions and approaches used to reduce anesthesia, increase compliance and improve the experience of pediatric cancer patients undergoing these types of procedures (40, 45, 53, 55–57).

**Figure 2:**
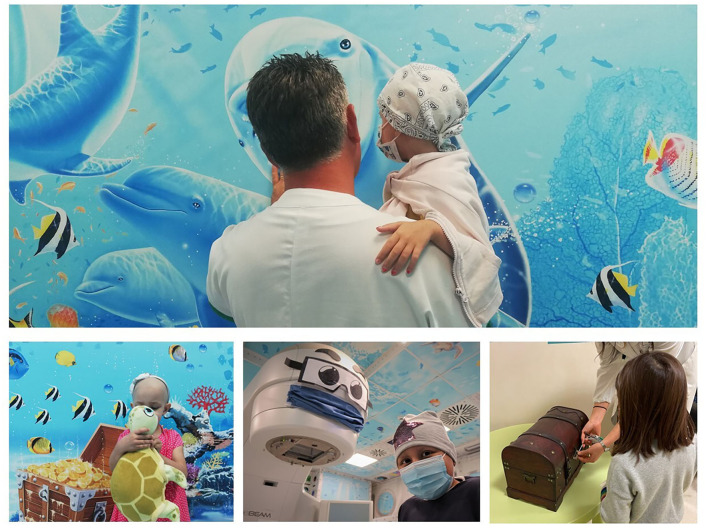
Pediatric radiotherapy, any RADAR pathway' images and tools.

Our Center has recently provided an annually average of 140 pediatric treatments. The care path based on a bio-psychosocial approach was carried out by a dedicated multidisciplinary team of doctors, nurses, technicians, psychologists, and anesthetists. The RADAR project was born to increase the personalization of pediatric RT through a multidimensional approach. The project found its inspiration from a marine setting reproduced by an artist on the walls of the treatment room (Figure 2).

The project uses assessment tools, such as the Multidimensional Assessment for Pediatric patients in Radiotherapy - MAP-RT schedule (58), age-appropriate psychological preparation and psychological support, creative activities, and digital tools. All these interventions increasingly try to put the patient/family at the center by fostering engagement and co-creation processes in RT.

Among RADAR's many activities, one of the most appreciated interventions by patients/parents is an intervention based on the principles of the token economy (59–61); this method has already proven its effectiveness in other contexts (62–64). Over the last 4 years, we have built an autonomous system on understanding the feasibility of a method based on the principle of reward and “reward” in RT.

We called it “the Dreams Chest,” and it offers pediatric patients the opportunity to choose online a present to receive on the last day of their therapy. According to this program, the daily RT sessions are considered a “token” to reach the treasure. The final goal represents the child's dream, although, within a fixed budget, the object takes on a personal value because it is personally chosen. More than 400 children had the chance to express their dreams through this project, thanks to many donors and large and small companies who paid for their gifts.

“The Dreams Chest” seems an economically sustainable method that can help increase adherence to RT in pediatric patients. Overall, since the start of the RADAR project, the use of anesthesia procedures has been significantly reduced, resulting in lower healthcare costs. Since 2018, when the experience began, the number of sedations fell from 19 to 13%, which in economic terms corresponds on average to 45.000€ per year. Among the childcare monitoring tools, and after translation and cultural Italian adaptation, we have subsequently included the Parents PedsQL™ Healthcare Satisfaction Hematology/Oncology Module, to assess the level of General Satisfaction, Information, Inclusion of Family, Communication, Technical Skills, and Emotional Needs.

***2019 - From needs to building***

### 2.4. MISSION: Multisensory Integrated SyStem for patIent cOmpliaNce improvement

**Figure 3:**
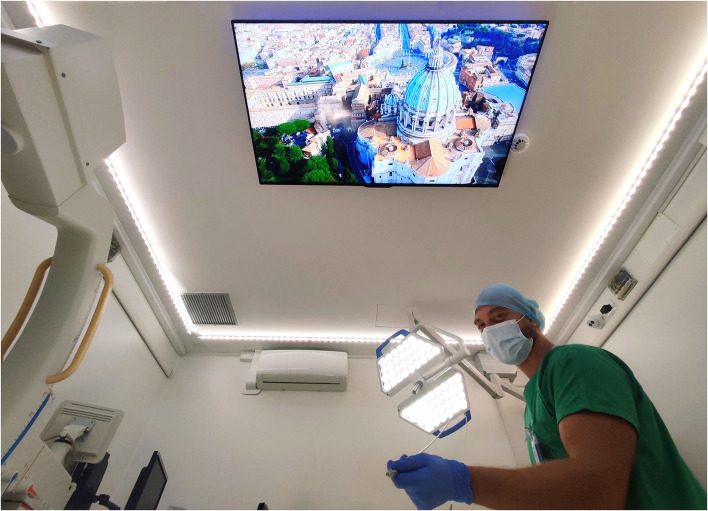
Interventional oncology center room (MISSION: Multisensory Integrated SyStem for patIent cOmpliaNce improvement).

The MISSION project was realized from the information that emerged both from the HAPPY protocol experience and from the feedbacks we received from patients Considering the results of the study, multisensory domotic equipment (sound/music, aromatherapy, chromotherapy, images) was subsequently installed in our Interventional Oncology Center IOC (Figure 3) to improve patients tolerance to treatments through a global approach (MISSION: Multisensory Integrated SyStem for patIent cOmpliaNce improvement). We are collecting preliminary results, but in clinical practice, the intervention is already proving effective in generating a widespread sense of calm and a better management of patients' anxiety.

***2020 – Gratitude staff members' intervention***

### 2.5. The digital group “Seeds of Gratitude”

In a person-oriented service, the needs of the people attending it are as important as those of the operators providing care. The aim is to enhance the aspects of the operator-patient relationship through attention to the relational and overall dimension of the person. Staff training courses and events on good interaction with patients are widespread; less attention is paid to interventions aimed to the wellbeing of groups and individual team members. The psychological wellbeing of healthcare workers in oncology has always been a critical issue due to the daily management of complex topics such as death and workload, resulting in a usually very high level of burnout (65). The impact of the COVID-19 pandemic on cancer patients was high in terms of anxiety, fear, and psychological distress (66). Medical staff from frontline wards, especially oncology units, was at increased risk of infection and burnout (67). Healthcare workers had to manage many challenges (68) and their psychological needs are increasingly important (69). In Italy, the emergency required timely interventions (70), especially in team working, which is crucial in multidisciplinary teams such as RT (71).

Literature in psychosocial sciences has shown that stress (72), fear (73), or emergency (74), influence human relationships; research shows that sense of belonging to the group and contact with others' emotions (75) play a central role in reducing these risks (76). Studies suggest that workplaces aiming to increase job satisfaction can do so through well-organized gratitude interventions (77). Gratitude is also related to wellbeing, and it can become helpful for healthcare professionals to relieve fatigue and restore meaning to their work (78). Therefore, during the lockdown, it was created a gratitude-focused “inter-group contact” tool (79) to increase group identity and mutual trust, rediscovering the pleasure of being part of a team. The project was conducted from April 2020, during the COVID-19 Italian lockdown.

**Figure 4:**
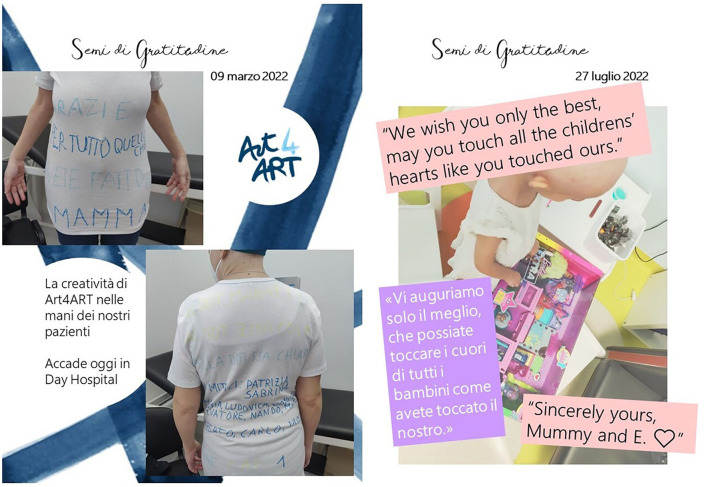
CTwo of 180 “Seeds of Gratitude” realized for the broadcast. Translation of the “Seeds of Gratitude”: “thanks for what you did for mum”; “the Art4ART creativity in the hands of our patients, it happens today in day hospital”; “we wish you only the best, that you may touch the hearts of all children as you have touched ours”.

This project consisted of a WhatsApp broadcast, in which a daily message mainly in JPEG format was published: creative cards composed of letters, emails, images, music, or videos accompanied by a short reflection (Figure 4). Patients' gratitude-oriented messages can help workers find a sense of gratification. In May 2020, when phase two started in Italy, the participants were surveyed on their satisfaction with the project (80). The results showed that 87.9 % of the staff members were satisfied with the experience (≥7 out of 10) and 89.6 % expressed that they would like to continue the experience; the activity is still active with one message per week. This experience could be extended to other units. The impact on the sense of cohesion and stress reduction could be investigated in the future. “Seeds of Gratitude” also became an online book (81).

***2021 - The art and technology paradigm in plan and platform***

### 2.6. The Art4ART project

Thanks to previous experience, the architectural renovation of one of the areas of the Gemelli ART led to the birth of the Art4ART Project (7), which was able to realize the renovation of the rooms starting from a theoretical framework. Thus, in 2021 the new Art4ART Unit was inaugurated.

In addition, the physical spaces of the center were collected into a web-based digital Art4ART platform. The platform provides patients with artistic content. It is not only an entertainment opportunity, but it represents a tool that allows emotional profiling of the patient. Data regarding patients' preferences and choices are stored and analyzed in a clinical research protocol also using AI algorithm to measure and predict impact indicators regarding:

- Patient compliance
- Clinical outcomes in terms of toxicity and survival outcomes
- Psychological profile among the several diseases.

Through the systematic acquisition of patient preferences and integration with other clinical parameters, some studies are ongoing to measure the clinical, psychological, organizational, and social impact of the Art4ART project. The use of digital technology will lead to the reversal of viewpoint from *therapeutic acts* to *patient-centered care*. Art4ART will offer an art-based digital supporting patients resilience and a research platform about the role of humanities as a cure in RT.

#### 2.6.1. The Art4ART project aims to

- Offer cancer patients undergoing RT the opportunity to enjoy several personalized artistic options to improve quality of life, compliance, efficacy, safety, and perceived quality of care.
- Use AI tools to profile patient preferences, integrate clinical data, and monitor through appropriate personalized interactions the use and benefit of the platform. This will be possible by administering on the platform psychometric psychological scales (7).
- Transform well-known international artworks and dedicate artists' productions to therapeutic tools for patients and dynamics exchanges with donors that would like to support assistance and research.

#### 2.6.2. The Art4ART project tools and metrics

**Figure 5:**
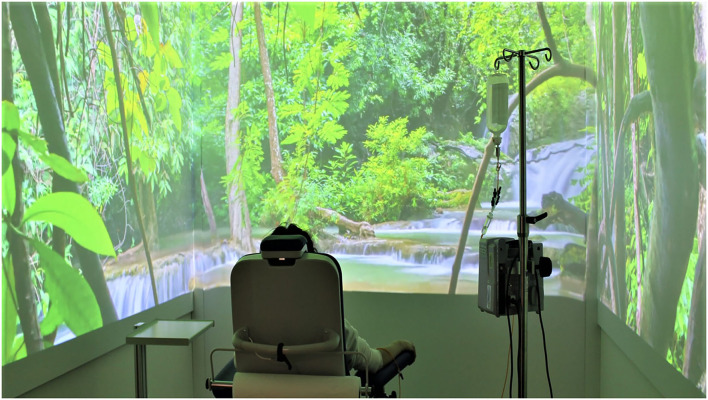
Multimedia immersive room use during chemotherapy.

**Figure 6:**
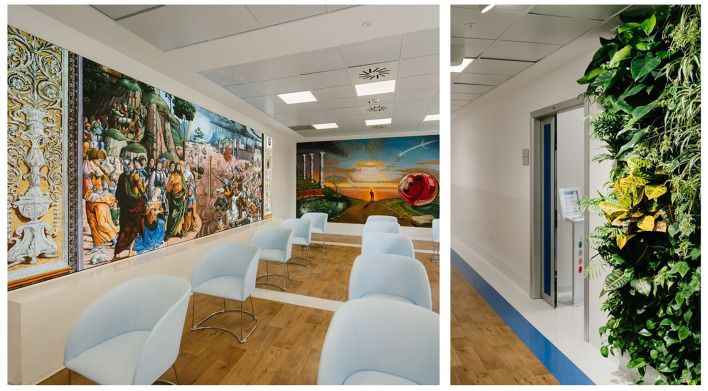
Details of the common areas of the Art4ART unit.

1. A web-based digital platform, Art4ART, has been developed to propose and share with patients several forms of art, such as video entertainment. Classifying each content according to eight human dimensions (friendship, love, attention, courage, self-care, enthusiasm, passion, and spirituality) and eight artistic channels (music, poetry, literature, cinema, nature, painting, sculptures, monuments, photography, profession).
2. A multimedia immersive room (Figure 5), where the patients during treatments can experience a 360° vision of video entertainment or several dedicated immersive experiences.
3. An art-based welcoming of the patients with an architectural and semantic metamorphosis of the treatment places: the concept of the *waiting room* has evolved toward a *welcome room* for patients called 'Odeon' according to the ancient Greeks' idea of art-dedicated theaters, with an 8-meters HD screen and a full-wall fresco painting (Figure 6). An ordinal number no longer identifies the chemotherapy infusion seats, but they are characterized by the name of a flower whose color they bear.

***2022 – Tailor-made interventions and applications***

### 2.7. Frail patients, the *Cristallo* project

Through the architectural renovation of the Art4ART inpatient unit and the combination of art and technology, we could design special care for frail patients in 2022. After the restrictions of COVID-19, considering the high psychopathological risk of the general population (82), we desired to pay attention to the physical and psychological impact of isolation and stress on cancer patients (83–85). Although the data are still few and partial, the oncology population, and their caregivers (86), are at risk of severe anxiety, stress, and depression (66, 67, 87) and long-term sequelae.

It is plain to see that different clinical conditions, treatment phases, and other clinical variables could encourage resources and coping strategies for oncological disease and therapies. In this scenario, the importance of a psycho-physical profile early assessment, especially in frail patients (older or with poor performance status) who have to undergo RT, could help healthcare professionals to identify high-risk situations and to perform a tailor-made treatment (88).

For these reasons, enhancing patient assessment and clinical monitoring during treatment reveals itself to be of essential importance. This type of early intervention could lead to the early identification of patients with possible psycho-physical frailties, personalization of care pathways, and supportive interventions, during hospitalization and after discharge.

One of the most discussed issues in the approach to the management of cancer patient is his frailty/complexity (89). By now, most new cancer diagnoses are made in patients over 70, where it is often possible to observe patients undertreatment or overtreatment (90). It is possible to keep a similar scenario in younger but frail patients or complex patients (91). Due to comorbidity, polypharmacotherapy, and social/economic network changes, those patients may have lower compliance to treatments or greater susceptibility to related toxicity treatments, The “Cristallo” project was in fact developed to overcome these problems and thanks to technological implementations in the environments This project focus on frail or complex patients for whom personalized management is essential. A therapeutic choice weighed on the patient's performance, a multidimensional approach to all comorbidities, and the patient's polypharmacy. The goal is not personalized but a tailor-made treatment designed for the patient in front of us. The Cristallo project uses a specific path from the outpatient clinic to any acute hospitalization. It follows the person with a new cancer diagnosis in various settings for cancer treatment, using geriatric oncology scores for the assessment and supportive care to manage related toxicity treatments.

**Figure 7:**
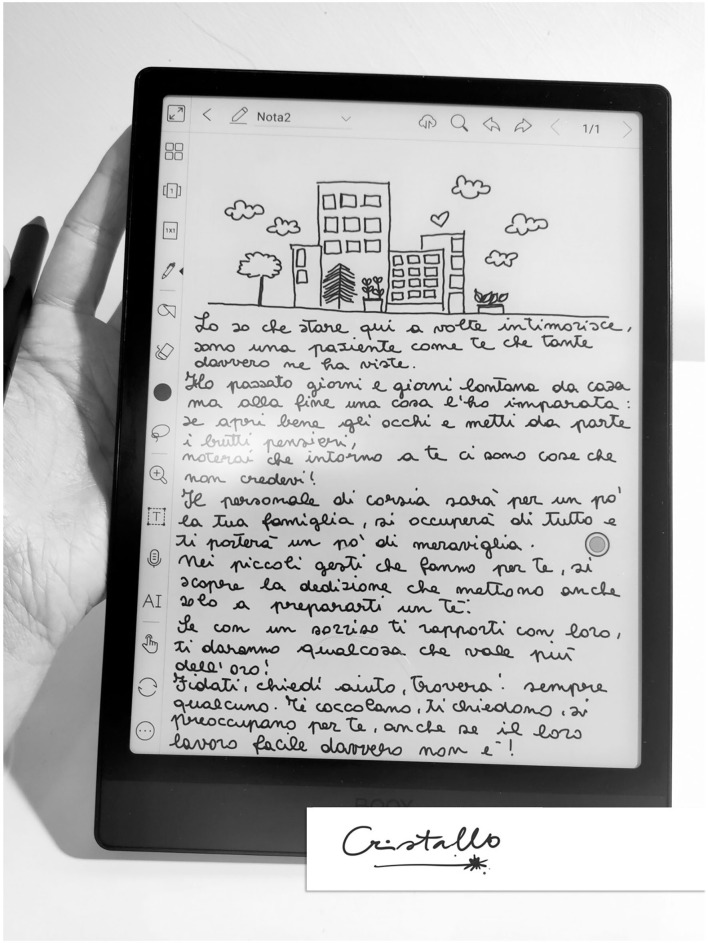
A patient's poem written on one of our Cristallo devices. Translation of the poem: “I know that being here is sometimes intimidating, I am a patient like you who has seen a lot. I spent days and days away from home, but in the end, I learned one thing: if you open your eyes wide and put aside your bad thoughts, you will notice that there are things around you that you did not believe! The ward staff will be your family for a while, they will take care of everything and bring you some wonder. In the small gestures they make for you, you will discover the dedication they put into even just making you a cup of tea. If you deal with them with a smile, they will give you something that is worth more than gold! Trust me, ask for help, you will always find someone. They pamper you, they ask you, they care for you even if their job is not at all easy!”.

In the Cristallo project scenario, a feature is represented by preserving the person's practical skills as objects contact or touch and maintaining self-sense. This is achieved through the use of graphic devices (Figure 7) that allow the preservation of one's proprioception, handwriting, and body perception.

## 3. Discussion

The implementation of art and digital technology in a value-based perspective can contribute to the mutual integration between cancer patient's pathway and valorisation of the patient's perceived value (92–95). The physician-doctor gap (96) has led us to reconsider in terms of “patient proximity” technological innovations such as data mining (97–101), process mining (102, 103), omics-based predictive models (104–113), patient telemonitoring, patients' communication and e-health (114, 115). These innovations can also enhance the patient's compliance and allow a better patient experience (80, 116). In several studies, including randomized clinical trials, the impact of digital technology for patient monitoring during oncological treatments was found to be effective in increasing survival outcomes (117, 118), in prevent recurrent emergency department visits (119, 120) and in improving patients physical functions, symptoms control and quality of life (121). The opportunity of reducing cancer-related toxicities represents an exciting perspective not only on the clinical-scientific and ethical side but also from an economic perspective. Indeed, cancer-related toxicity costs' are well known in literature (122). Ashmore et al. emphasized that digital technology should also be used to maintain contact with the facility also between the end of care and the subsequent follow-up when the toxicity may occur or exacerbate (123). This participatory design and co-creation mode is also considered central to ensuring equity in digital health intervention (124, 125). Bhargava et al. (126) confirmed the possibility of cost reduction through digital technology introducing a digital remote symptoms self-reporting application. The economic saving was over 62,000 dollars in a pilot study with 13 patients affected by cancer and receiving palliative care (126).

In our Center, structural, logistic, and psychological interventions merging art and technology have been implemented in the last years. This experience integrates digital technology and the beauty of art into the standard of cancer care to provide a holistic approach to cancer patients.

The use of art therapy in cancer care is a strategy that has been explored in recent years (13, 15, 18). Although there is no conclusive evidence of improved survival outcomes on randomized clinical trials (13, 18), the feedback from patients in small case series makes us consider this approach interesting (15). The interoperability between personalized artistic proposal offered through digital devices and the hospital's electronic medical records (EMR) give us the possibility to include also those data in our clinical studies. To date, the introduction of art as a communication channel has raised awareness among supporters and sponsors of our Center.

Digital technology, like every technological innovation introduced in the healthcare sector, also requires dedicated education and training of both healthcare and administrative staff (127–129). The effectiveness of digital education interventions in different health care disciplines has been recently reviewed (130). Most studies focused on health professions education in general, surgery, and nursing. The main modalities are virtual reality and online education (130).

Recently, based on the need for an ethics evaluation that keeps the person at the Center of each technology, a new topic has been introduced, namely *algor-ethics*. It approaches the view of considering new technologies as tools for humans and preventing the possibility that they become opportunities for imbalance, disparity, or even damage.

The decision to dedicate technological innovations to support the patient in the treatment pathway by synergizing communication with the patient can open the frontiers of a “new digital humanism”. In this “new humanism” technology represents a tool in support of the “human part” (131–133) opening the field of the human guided digital health in oncology.

The integration of digital technology with artistic proposal during medical and radiation oncology treatments is the fascinating challenge we decided to take on. Next steps and future perspectives are represented by the systematic measure of the impact of such technology in term of clinical outcomes.

## Data availability statement

The raw data supporting the conclusions of this article will be made available by the authors, without undue reservation.

## Ethics statement

The studies involving human participants were reviewed and approved by the Comitato Etico, Fondazione Policlinico Universitario Agostino Gemelli IRCCS, Rome Italy. Written informed consent to participate in this study was provided by the participants' legal guardian/next of kin.

## Author contributions

CC, LD, VV, MG, and LT proposed the work. CC, EM, LD, PC, and SS organized and collected the material and wrote the first draft of the manuscript. SC, FB, and SB revised and corrected the draft. CC, LD, EM, PC, and GC wrote supplementary sections of the manuscript. GC, DC, LT, VV, and MG revised the last version of the manuscript and offered clinical experience in the field. AS contributed to the language review. All authors contributed to manuscript revision, read, and approved the submitted version.
